# Soft Tissue Dehiscence Associated with a Titanium Patient-Specific Implant: A Prosthetic Solution as an Alternative to Soft Tissue Grafting

**DOI:** 10.1155/2021/5125375

**Published:** 2021-12-21

**Authors:** Sharaf Eldeen M. Abbas, Mohamed A. ELKhashab

**Affiliations:** Department of Prosthodontics, Faculty of Dentistry, Cairo University, Cairo, Egypt

## Abstract

*Patients*. This clinical report describes the detailed prosthodontic management of a 23-year-old male patient suffering from soft tissue complication following the placement of a 3d-printed titanium patient-specific implant. This implant was implemented simultaneously with the resection of a calcifying cystic odontogenic tumor related to the maxillary arch. Later, soft tissue dehiscence and implant exposure were encountered with subsequent food impaction, infection, and pus discharge. The treatment plan was to fabricate removable partial denture. The prosthesis was planned to be retained by bar and clip attachment on the patient-specific implant side, while on the other side, the removable prosthesis was allowed to engage two abutments with an embrasure clasp assembly in addition to covering the palatal tissues to offer protection for the soft tissue dehiscence against food impaction. *Discussion*. Soft tissue dehiscence and implant exposure are among the frequently reported complications associated with the patient-specific implant. The resulting infection complicates the prognosis of the implemented implant and necessitates, in some occasions, its removal. The selection of the removable prosthesis to cover soft tissue dehiscence was a conservative alternative to the implant removal as it protects the exposed titanium surface from food impaction while maintaining the implant functionality. *Conclusion*. Three-year follow-up showed complete resolution of the patient's complaints while fulfilling the patient's aesthetic and functional demands and indicates that the use of detachable overlay prosthesis could be one of the proposed treatment options.

## 1. Introduction

For the past decade, facially anchored osseointegrated implants [1] and free flaps [2] have been universally accepted for maxillofacial reconstruction after surgical resection. Such advances in surgical reconstruction techniques have brought up the possibility of complete defect closure and provide a good foundation for further intraoral implant placement, allowing the patients to experience more life-like restorations [3–5]. Unfortunately, because of the insufficient bone after tumor ablation surgeries, these reconstructive procedures often require demanding surgeries which are associated with distant site morbidity and prolonged healing period [3].

Thanks to the advances in imaging modalities and manufacturing techniques, patient-specific implants (PSIs) have been proposed to overcome the pitfalls associated with the previously mentioned treatment options [6]. Initially, PSI was fabricated by the prebending of metal plates over 3d-printed models [7]. Unfortunately, with this technique, PSI suffered from metal plate weakness following bending [8, 9]. Recently, patient-specific implants have been manufactured by 3d printing of metals with remarkable improvement in their strength and adaptation [6, 10–12].

Ciocca and his colleagues in 2012 [10] were the first to use 3d-printed PSI manufactured by direct laser metal sintering for mandibular reconstruction in conjunction with a free flap. Since then, several authors have reported the utilization of 3d-printed PSI for the restoration of maxillary and mandibular defects, alone or in conjunction with free flaps [12–14], using different materials such as titanium and polyether ether ketone (PEEK) [15].

Despite all attempts to enhance bone and soft tissue healing around PSI restorations, soft tissue dehiscence and recurrent infection have been reported in multiple studies [12, 16, 17]. The problem with these complications is the difficulty in management and recurrence that may necessitate, in some cases, the removal of the whole assembly [12]. In this case report, the authors describe a prosthetic solution to salvage a successfully anchored PSI associated with extensive soft tissue dehiscence.

## 2. Methodology

A 23-year-old male patient was referred to F.G. Maxillofacial Prosthodontics Unit, Faculty of Dentistry, Cairo University, after undergoing surgical installation of patient-specific implant (PSI) following surgical resection of calcifying cystic odontogenic tumor CCOT related to the left maxillary arch extending to the orbital floor. The patient was complaining of hygiene-related complications as a result of extensive soft tissue exposure of PSI.

Upon reviewing the patient's records, it was revealed that the patient was scheduled for total maxillary resection with simultaneous reconstruction using patient-specific implant prosthesis. With the aid of digital software (Materialise Mimics, Leuven, Belgium), the lesion was virtually resected, and the patient-specific implant was designed with maximum extensions engaging the whole maxillary surfaces superiorly, medially, and laterally. Finally, the implant was printed with an EBM machine (Arcam AB, Stockholm, Sweden) into medical grade 23 titanium Ti–6Al–4V, after then it was subjected to several procedures of surface treatment and sterilization.

At the time of tumor ablation surgery, the implant was installed and stabilized using 2 mm grade 5 titanium fixation screws on the facial bones extending from the orbital floor to the palate. The palatal soft tissue on the tumor side was preserved and used as a soft tissue drape to cover the implant surface. The patient did not receive any adjunctive treatment before or after the surgery. Three months after the surgery, the patient received a five-unit fixed partial denture restoration cemented on the extruding metal posts.

Six months later, the patient presented with soft tissue dehiscence and complained of frequent food impaction and persistent foul odor. The patient was instructed to rinse daily with antiseptic mouthwash till the condition subsides in addition to strict routine oral hygiene measures. By the age of 23, implant surface exposure has become more extensive, and the patient suffered further hygiene-related complications. Accordingly, the surgical team decided to remove the PSI. However, based on the patient's desire not to get involved in further surgical interventions, he was referred to our team for possible prosthetic management.

**Figure 1:**
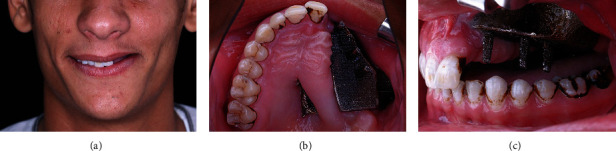
Initial situation when the patient presented to the prosthodontic clinic: (a) extraoral frontal view, (b) intraoral occlusal view, and (c) intraoral left side view.

Data collected from patient interviewing, clinical examination, and radiographic imaging revealed that PSI was stable, functioning, and pain-free. Titanium surface was exposed and extending along the buccal and palatal surfaces of PSI (Figure 1).

Diagnostic impressions were made for both arches by using irreversible hydrocolloid impression material (Cavex CA37 normal set, Haarlem, Netherlands). A customized maxillary tray was fabricated by using cold cure acrylic resin (Acrostone, cold cure special tray material, Cairo, Egypt) over which the definitive impression was made by using a two-step impression technique with rubber base impression material (Zhermack, Zetaplus & Oranwash VL, Badia Polesine RO, Italy). Inlay pattern resin (Duralay, Reliance, Edmonton, Canada) supported with metal wires was used to pour the metal posts portion of the impression while the rest was completed with a hard stone. Modeling wax (Cavex, Set Up Hard, Haarlem, Netherlands) occlusion rims were adjusted intraorally over cold-cured acrylic resin record bases, and the interocclusal record was made at maximum intercuspation. Casts were mounted on a semiadjustable articulator with a face bow transfer. Wax-up of acrylic teeth was done for diagnostic and treatment planning purposes.

A condensation silicon putty index was adapted on the buccal aspect of the diagnostic wax-up to determine the prosthetic space available occlusocervically and buccolingually, the size of the artificial teeth, and its relation to the metal posts. A 20 mm interocclusal distance was revealed with sufficient space between the metal posts and the buccal aspect of the future teeth. Surveying of the cast was carried out to determine the amount of natural teeth undercuts, guiding planes, and the potential path of insertion.

Accordingly, the plan was to exclude the middle post and fabricate a bar and clip attachment connecting the two distant metal posts to support a partial overdenture. Embrasure clasp assembly was planned on the maxillary right first molar and maxillary right second premolar to aid for retention, support, and bracing in addition to a palatal plate extending over the palatal surfaces of the maxillary right first premolar, canine, lateral incisor, and central incisor and maxillary left central incisor to the areas of soft tissue dehiscence at the patient-specific implant. Before the execution of the planned prosthetic management, the patient was instructed to follow comprehensive oral hygiene measures. These instructions included rinsing with chlorhexidine mouthwash twice daily and brushing the PSI with soft toothbrush and chlorhexidine gel. Besides that, he was encouraged to use dental floss between the remaining teeth and tooth brushing by soft toothbrush twice daily. Empirical antibiotics were prescribed to the patient to manage any possible infection at the exposure site.

**Figure 2:**
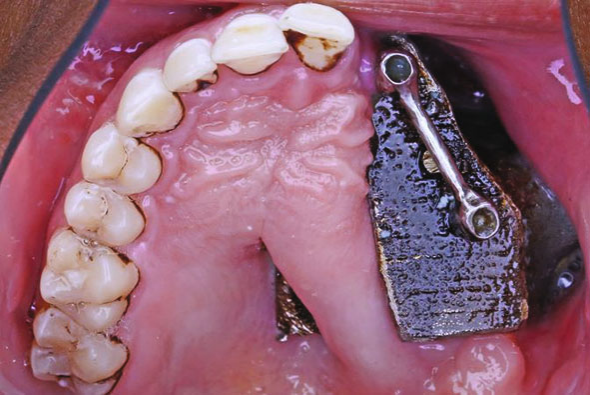
Intraoral occlusal view showing the bar after cementation.

**Figure 3:**
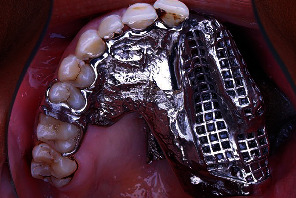
Intraoral occlusal view showing metal framework try-in.

The planned mouth preparation was executed as follows: cutting the middle post by using a transmetal bur under copious irrigation, preparation of a mesial occlusal rest seat on the maxillary right first molar, and preparation of a distal occlusal rest seat on the maxillary right second premolar. Additionally, there is a mesial guiding plane on the proximal surface of the maxillary left central incisor. The definitive impression and mounting procedures were performed with the same techniques mentioned earlier. OT Bar multiuse (Rhein83, Bologna, Italy) was waxed, cast into Co-Cr, and then cemented on the metal posts (Figure 2). Metal framework partial denture was cast and tried intraorally to check for the fit, stability, support, retention, and path of insertion (Figure 3).

The trial setting of artificial acrylic teeth guided with the diagnostic wax-up was done and tried intraorally. The approved wax trial tooth arrangement was flasked and packed by using heat-cured acrylic resin (Acrostone, heat cure denture base material, Cairo, Egypt). At the time of bar and clip attachment pickup, the undercut beneath the bar was blocked out using a putty consistency rubber base to avoid the unnecessary flow of the pickup material. After insulating the polished surface of the prosthesis, a doughy mix of cold-cure acrylic resin was added to the intaglio surface, and a medium retention clip was picked up under maximum intercuspation.

**Figure 4:**
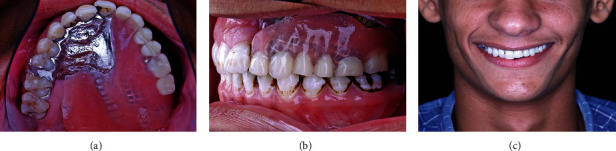
After prosthesis insertion: (a) intraoral occlusal view, (b) intraoral left side view, and (c) extraoral frontal view.

The prosthesis was finished and polished, and the patient received instructions regarding prosthesis insertion, removal, and maintenance (Figure 4). Additionally, strict daily oral hygiene measures were instructed to the patient. These instructions include tooth brushing with soft toothbrush daily, cleaning the prosthesis and PSI immediately after food ingestion, and rinsing with chlorhexidine for 14 consecutive days monthly.

**Figure 5:**
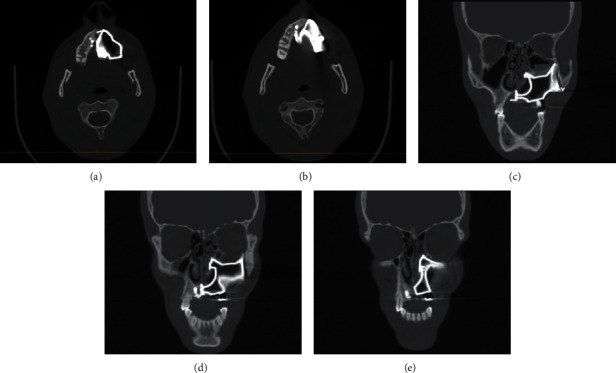
Computed tomography after 3 years of follow-up: (a, b) axial cuts and (c–e) coronal cuts.

The patient was recalled monthly for the first year and every three months during the following years. During this period, the patient received professional intraoral cleaning and debridement every 3 months. After 3 years of follow-up, no signs or symptoms of hygiene-related complications were observed clinically or radiographically (Figure 5). Additionally, the patient reported complete resolution of previously mentioned complaints as well as significant satisfaction with the aesthetics and function of the prosthesis.

## 3. Discussion

Surgical reconstruction is nowadays considered a well-accepted treatment modality for the rehabilitation of oral defects, and it is achieved either by using free grafts, zygomatic implants, or patient-specific implants. Advances in volumetric imaging and 3d planning before the ablation surgery allow the surgeons to make more accurate decisions regarding the procedure and give the chance to manufacture personalized implants with the help of additive manufacturing technology to fit more readily during surgery.

In this patient, the implant was 3d printed from Ti6Al4V. The surfaces of PSI facing the bone were subjected to sandblasting and chemical treatment procedures in an attempt to enhance osseointegration, while the surfaces facing the soft tissue were left unpolished after the 3d printing procedure. Although it was hypothetically claimed that the unpolished surface facing the soft tissue would decrease the possibility of its dehiscence [11], our patient suffered from extensive soft tissue dehiscence with subsequent food impaction and bad odor. This sort of complication has been documented previously by several authors [12, 16, 17]. Rendenbach et al. [17] studied the placement of PSI in conjunction with free flaps and reported plate exposure in 29.7% of CAD/CAM plates. Similarly, Mounir et al. [12] described the removal of three mandibular PSIs placed immediately following benign tumor removal due to failure of management and resolution of infection.

Despite the several attempts to manage the condition by medication, debridement, and daily oral hygiene measures, the soft tissue complications were persistent and bothersome to the patient. Custom implant removal was the first line of treatment proposed by the surgical team. Accordingly, an immediate restoration of the resultant surgical defect was addressed either using immediate surgical obturator or surgical reconstruction. Several authors [5] have suggested the use of osteomyocutaneous free graft with subsequent implant placement as a reliable option which allows implant insertion using flapless or open flap technique. An alternative conservative prosthetic option was recommended by the prosthetic team based on the patient preference to overcome the soft tissue dehiscence problem and eliminate the need for implant removal. The removable prosthesis was selected for this case to fully cover the remaining palatal tissues and the area of soft tissue defect at the implant site to avoid food impaction during function and to allow full access for proper oral cleaning procedures. Retention and support of the prosthesis were primarily derived from PSI and the remaining teeth with no support derived from the soft tissue.

On the intact side, the removable prosthesis was allowed to engage two abutments with an embrasure clasp assembly in addition to covering the palatal tissues to offer cross arch stabilization. On the implant side, the presence of enough prosthetic space enabled the use of a bar and clip attachment, which fulfilled the high retention demands in comparison to other attachment systems [18].

The main limitation associated with the chosen treatment option is the lack to provide a permanent soft tissue closure but rather preventing food impaction which is highly dependent on the patient compliance. The patient was instructed to follow strict oral hygiene measures; however, it was not anticipated to experience significant food impactions beneath this rigid design unlike soft tissue-supported ones. Another addressed concern is the need for continuous follow-up clinically and radiographically to ensure the patient's compliance and to detect early signs of possible complications. The possibility of discarding the prosthesis and the removal of the PSI was also discussed with the patient in case of the development of persistent infection, which could be life threatening.

## 4. Conclusion

Patient-specific implants may be regarded successful in terms of fixation and stability; nevertheless, soft tissue dehiscence is a serious complication that should be anticipated and managed early during the treatment. The use of detachable overlay prosthesis can be considered a promising solution to conservatively overcome the hygiene-related complications while fulfilling the patient's aesthetic and functional demands.

## Data Availability

The datasets generated and/or analyzed during the current case report are available from the corresponding author (Sharaf Eldeen M. Abbas) on reasonable request.
